# Cross-sectional assessment of prevalence and correlates of blood-borne and sexually-transmitted infections among Afghan National Army recruits

**DOI:** 10.1186/1471-2334-12-196

**Published:** 2012-08-21

**Authors:** Catherine S Todd, Abdul Nasir, G Farooq Mansoor, Sayed M Sahibzada, Linda L Jagodzinski, Farzana Salimi, M Naim Khateri, Braden R Hale, R Vincent Barthel, Paul T Scott

**Affiliations:** 1 Department of Obstetrics & Gynecology, Columbia University, 622 West 168th Street PH 16-69, New York, NY, 10032, USA; 2 Health Protection and Research Organization, House P-860, Street 10, Taimany, Kabul, Afghanistan; 3 U.S. Military HIV Research Program, Walter Reed Army Institute of Research, 503 Robert Grant Avenue, Silver Spring, MD, 20910, USA; 4 Afghan Public Health Institute, Ministry of Public Health, Masoud Circle, Kabul, Afghanistan; 5 Ministry of Defense, Shash Darak, Kabul, Afghanistan; 6 Department of Defense HIV/AIDS Prevention Program, Naval Health Research Center, Naval Health Research Center, 140 Sylvester Rd, San Diego, CA, 92106-3521, USA; 7 U.S. NAMRU-3, Ramses Extension Street near Abbasia Fever Hospital, Cairo, Egypt; 8 U.S. Military HIV Research Program, Walter Reed Army Institute of Research, 503 Robert Grant Avenue, Silver Spring, MD, 20910, USA

**Keywords:** Afghanistan, Military populations, HIV, Sexual risk behavior, Drug use

## Abstract

**Background:**

Few data are available in Afghanistan to shape national military force health practices, particularly with regard to sexually-transmitted infections (STIs). We measured prevalence and correlates of HIV, syphilis, herpes simplex 2 virus (HSV-2), and hepatitis C virus (HCV) among Afghan National Army (ANA) recruits.

**Methods:**

A cross-sectional sample of male ANA recruits aged 18–35 years were randomly selected at the Kabul Military Training Center between February 2010 and January 2011. Participants completed an interviewer-administered questionnaire and serum-based rapid testing for syphilis and hepatitis C virus antibody on-site; HIV and HSV-2 screening, and confirmatory testing were performed off-site. Prevalence of each infection was calculated and logistic regression analysis performed to identify correlates.

**Results:**

Of 5313 recruits approached, 4750 consented to participation. Participants had a mean age of 21.8 years (SD±3.8), 65.5% had lived outside Afghanistan, and 44.3% had no formal education. Few reported prior marijuana (16.3%), alcohol (5.3%), or opiate (3.4%) use. Of sexually active recruits (58.7%, N = 2786), 21.3% reported paying women for sex and 21.3% reported sex with males. Prevalence of HIV (0.063%, 95% CI: 0.013- 0.19), syphilis (0.65%, 95% CI: 0.44 – 0.93), and HCV (0.82%, 95% CI: 0.58 – 1.12) were quite low. Prevalence of HSV-2 was 3.03% (95% CI: 2.56 - 3.57), which was independently associated with age (Adjusted Odds Ratio (AOR) = 1.04, 95% CI: 1.00 - 1.09) and having a television (socioeconomic marker) (AOR = 1.46, 95% CI: 1.03 – 2.05).

**Conclusion:**

Though prevalence of HIV, HCV, syphilis, and HSV-2 was low, sexual risk behaviors and intoxicant use were present among a substantial minority, indicating need for prevention programming. Formative work is needed to determine a culturally appropriate approach for prevention programming to reduce STI risk among Afghan National Army troops.

## Background

Military populations provide an accessible and often nationally-representative sample for disease surveillance that shapes vaccination policy [1,2]. Screening for other infectious diseases is more problematic, as policy and programmatic responses are less clear. Many militaries have policies to perform verbal or serological screening for HIV, with affirmative/reactive results constituting an exclusion criterion [1,3]. However, risk for HIV and other sexually-transmitted infections (STIs) persists following conscription with measurable prevalence of these infections detected among active duty troops [4-8]. Programming responses to mitigate STI risk need to be contextually appropriate and data-driven, with few published interventions tailored to military personnel [3,9,10].

In Afghanistan, the government is working to establish infrastructure and a trained deployable civil defense organization, the Afghan National Security Forces (ANSF), composed of the Afghan National Army (ANA), Afghan National Police (ANP), Afghan National Air Corps, Afghan National Security Police (intelligence service), and National Border Police. Troop recruitment, training, and retention activities are underway and command substantial foreign financial and human resource investment [11]. Target recruit populations are predominantly males aged 18 to 35 years, drawn from a country with an estimated literacy rate of 43.1% for males aged 15 years or greater [12]. Current ANA policy states that troops receive medical screening and care and that HIV-infected individuals may not enlist; however, screening for HIV or other infectious diseases is not routinely performed. Further, though medical care is provided through the military health system, there is little public health or prevention programming available. Though there are strong cultural proscriptions against use of intoxicants, a recent report noted positive urine screening tests for marijuana and opiates among 12 to 41% of ANP recruits [13]. The same duality may apply to sexual risk behaviors, for which there is little data among ANSF troops. The purpose of this study is to determine prevalence and correlates of HIV, syphilis, herpes simplex-2 (HSV-2), and hepatitis C and describe risk behaviors among ANA recruits.

## Methods

### Setting

**Figure 1:**
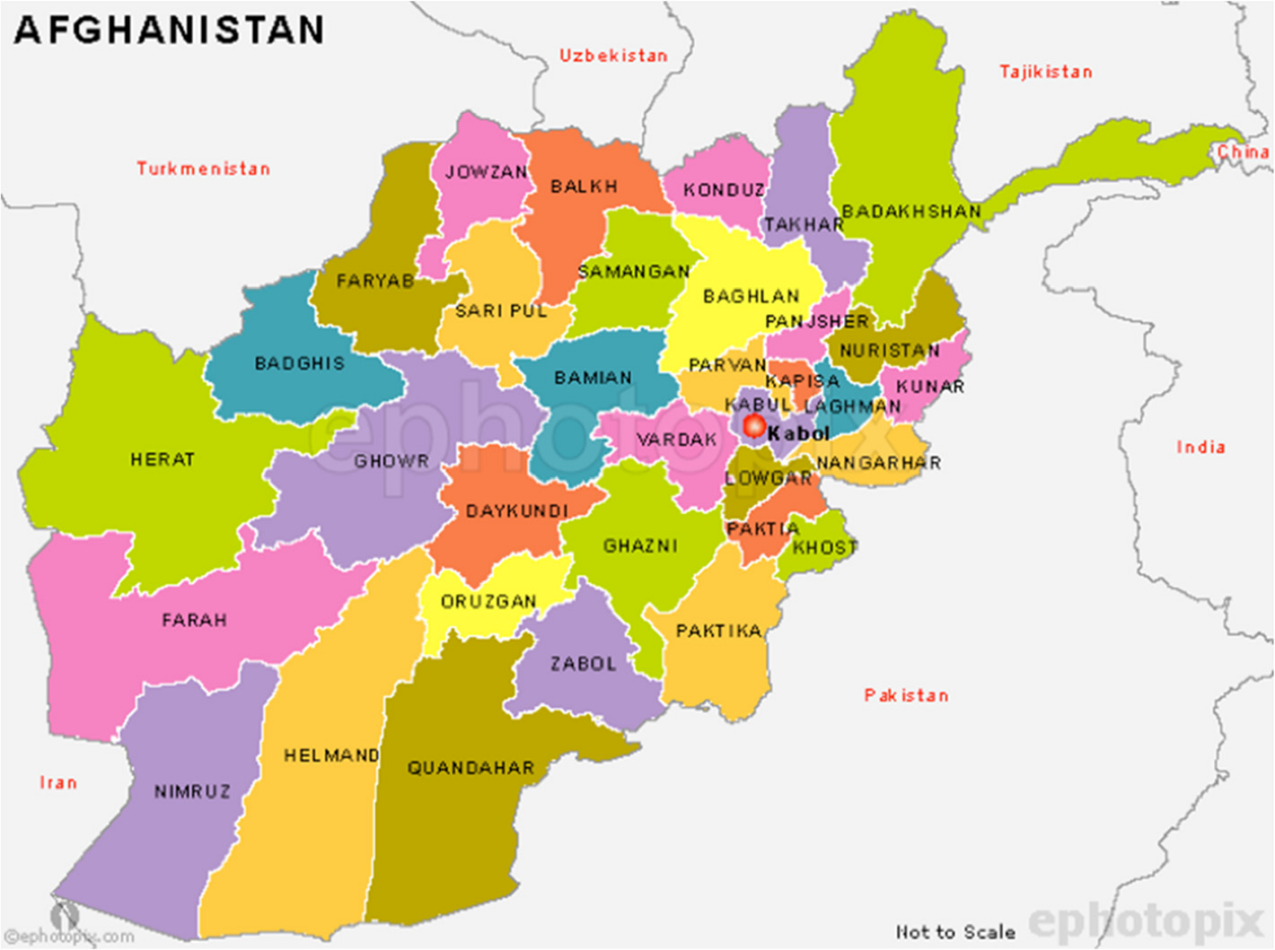
Map of Afghanistan

The study was conducted at the Kabul Military Training Center (KMTC), a large installation serving as a central point for recruit and officer education. At the time of the study, potential ANA recruits arrived at KMTC for medical screening and basic training exercises, remaining up to one month to allow sufficient time for *kandak* (unit) recruitment, comprising 2000 troops. Medical screening, consisting of a urine intoxicant test, vision and biodata evaluation, and physical examination, is the first activity all recruits undergo; those deemed unfit stayed for at least two days before returning to their home provinces. Of potential recruits presenting for ANA entry, approximately 90% conscript. The key reasons for ineligibility to join the ANA are failed urine drug screen for opiates (20%), physical disability (20%), age outside of required range (18–40 years) (15%), eye disease or vision impairment (15%), and mental illness or epilepsy (10%) (personal communication, Dr. Sayed Zaman). During the study period (February 2010 to January 2011), approximately 50 to 800 potential recruits were present daily. During study enrollment, regional recruitment centers were not routinely performing screening and intake, necessitating most recruits to transit through KMTC (Figure 1).

### Study design and participants

For this cross-sectional study, 27 potential participants, the maximum for study team capacity, were randomly selected from the recruitment pool on all working days. Eligible participants were those aged 18 years or greater, able to speak Dari or Pashto, and able to provide informed consent. Approval was obtained from the institutional review boards of the Columbia University, the Walter Reed Army Institute of Research, and the Ministry of Public Health (MoPH) of the Islamic Republic of Afghanistan.

### Measures

The questionnaire elicited demographic, socioeconomic, and migratory information from participants, with time spent inside and outside of Afghanistan quantified in months. Medical history and lifetime STI symptoms were assessed. Risk behaviors of interest included lifetime and current intoxicant use and sexual history, including sexual contact with female sex workers (FSWs), men having sex with male partners (MSM), and condom use.

### Procedures

Study staff enumerated recruits presenting for medical screening each day. Corresponding randomization numbers were then generated and the designated recruits approached by study staff and invited to an information session. Following general information, those willing to participate were taken individually to private rooms and provided informed consent. Those declining study entry or ineligible were asked to complete a brief demographic screening tool. Consented participants completed pre-test counseling and intravenous sampling. The study representative then administered the questionnaire, followed by post-test counseling and provision of syphilis and HCV Ab results.

### Serologic Testing and Follow-Up

All rapid testing was performed with SD Bioline kits (Standard Diagnostics, Kyongi-Do, Korea); testing for antibodies to hepatitis C (HCV Ab) and syphilis were performed at the study site, while HIV and HSV-2 screening and all confirmatory testing were performed at the Afghan Public Health Institute laboratory. HSV-2 screening was performed with HerpeSelect HSV-2 ELISA (Focus Technologies, Cypress, CA); repeat testing in duplicate/triplicate (at least two clearly positive tests of three runs) was used for confirmation. All reactive rapid tests received confirmatory testing, with Western Blot (LAV I/II Blot, BioRad, Redmond, WA) used to confirm HIV. Polymerase chain reaction (PCR) (Abbott RealTime HCV, Abbott Molecular Diagnostics, Des Plaines, Illinois, USA) was performed as the initial confirmatory test for HCV. This choice was prompted by cost; the price for each sample for PCR was 20% of the cost of the reflex recombinant immunoblot assay (RIBA) (Chiron RIBA 3.0 SIA, Chiron Company, Emeryville, California) assay. Non-viremic samples were then tested for hepatitis C antibody with RIBA. *Treponema pallidum* plasma agglutinin assay (TPPA) (Fujirebio, Wilmington, DE, USA) was used to confirm syphilis infection, followed by rapid plasma reagin (RPR) titre for clinical disposition.

Participants were encouraged to return within one week to the study site for screening and confirmatory results; no effort was made to trace individuals with positive test results as no personal identifiers were collected from the outset to preserve confidentiality. Participants were required to present their study number card to obtain results. Treatment referrals were provided for all participants with confirmed cases of hepatitis C and HIV; participants with confirmed syphilis were given the option for on-site treatment with benzathine penicillin or referral to a public sector clinic.

### Analysis

Descriptive statistics were generated for the study population. Student’s *T*-test and the binomial probability test were used to compare participants and those ineligible or declining entry. Prevalence for each infection was calculated with confidence intervals based on Poisson distribution. Correlates of sexual risk behaviors and HSV-2 were assessed with logistic regression analysis. Data were analyzed with Stata 11.0 (Stata Corp, College Station, Texas, U.S.).

## Results

Of 5313 recruits approached between February 2010 and January 2011, 4750 consented to participation; 54 were excused due to not speaking Dari or Pashto, 12 excused due to age ineligibility, and 497 declined entry, resulting in a 10.5% refusal rate. Non-participants were significantly more likely to have been born in Afghanistan (96.3% vs. 81.7%, p < 0.01) and differ by province of origin (p < 0.01).

Participant characteristics are summarized in Table 1. Generally, participants were young, had little or no formal education, and many had previously lived outside Afghanistan. Approximately one-fifth (19.8%) originated from Nangahar Province. Many participants reported having a radio in the home, while having a television or private family vehicle or eating meat more than twice weekly was uncommon. As a marker for access to medical care, the majority (91.5%, N = 4345) had seen a physician in their lifetime, of whom, 2350 had seen a medical provider between one and three times in the last year and 607 had seen a provider three or more times. Nearly one-third (29.1%) reported history of depression or anxiety, of whom 8.0% (110) reported currently taking medications for these conditions.

**Table 1:** Sociodemographic and migration characteristics among a cross-sectional sample of male Afghan National Army recruit participants, 2010–2011 (N = 4750)

**Variable**	**Mean** **±** **SD**	**Median**	**Interquartile range**
Age (years)	21.82 + 3.77	20	18 – 24
Number in Household	11.2 + 7.72	10	7 – 13
Time Lived Outside			
Afghanistan (months)	90.1 + 83.3	60	19 – 144
Time Following			
Repatriation (months)	54.9 + 59.4	36	8 – 86
	Number	Percentage	
Country of Birth:			
Afghanistan	3882	81.7%	
Pakistan	791	16.7%	
Iran	75	1.6%	
Other	2	0.04%	
Province of Origin (of 3882 born in Afghanistan):			
Non-Nangahar	3113	80.2%	
Nangahar	769	19.8%	
Ever Lived Outside Afghanistan:			
Yes	3110	65.5%	
Countries of Residence (more than one answer permitted):			
Pakistan	2172	69.8%	
Iran	1139	36.6%	
United Arab Emirates	40	1.3%	
Other	72	2.3%	
Civil Status:			
Never Married	2903	61.1%	
Currently Married	1831	38.5%	
Widowed	14	0.3%	
Divorced	2	0.1%	
Level of Education:			
None	2105	44.3%	
One year up to primary	1019	21.5%	
Some secondary school	903	19.0%	
Completed secondary or higher	723	15.2%	
Site of Education (N = 2645):			
Afghanistan	2237	84.6%	
Pakistan	507	19.2%	
Iran	68	2.6%	
Employed Prior to Recruitment:			
Yes	1140	24.0%	
Frequency Family East Meat Weekly:			
None	1173	24.7%	
Once	1647	34.7%	
Twice	1212	25.5%	
More than Twice	715	15.1%	
Usual Mode of Transportation:			
Taxi	559	11.8%	
Family’s Car	391	8.2%	
Bus/TownAce	1397	29.4%	
Animal-drawn cart	28	0.6%	
Motorcycle	95	2.0%	
Foot	2276	47.9%	
Radio in Home:	3288	69.2%	
Television in Home:	1552	32.7%	

Prior incarceration was reported by 16.4% (N = 780), of whom 10.3% had been incarcerated more than once and 30.1% were incarcerated in other countries. The most common reasons for incarceration were illegal entry or expired visa/passport in other countries (N = 227), fighting (N = 225), alleged work for Taliban (N = 20) or government (N = 22), and drug cultivation or trafficking (N = 41). Of those previously incarcerated, 11.2% (N = 87) had used intoxicants in prison.

### *Risk Behaviors*

Intoxicant use and sexual risk behaviors were assessed. Tobacco use was common, with *naswar* (snuff) and cigarette use reported by 1246 (26.2%) and 979 (20.6%) participants, respectively. Hashish (marijuana) use was also frequent with 775 (16.3%) reporting use, of whom 235 (30.3%) were daily users and initiated use at 17.9 (SD ± 3.7) years. Only 249 (5.3%) reported prior alcohol use, of whom 3% reported daily use. Other intoxicant use was uncommon, as only 160 (3.4%) reported regular opium use, 38 (0.8%) regular psychotropic or pharmaceutical analgesic use, and 23 (0.5%) reported regular heroin use. Of opium and heroin users, 16.9% and 56.5% reported daily use and initiated use at 19.3 (SD ± 4.4) and 20.4 (SD ± 4.3) years, respectively. Only three participants had ever injected drugs, of whom, only one reported current injection use.

Of 4729 respondents, 58.9% (N = 2786) reported prior sexual activity with a mean number of 1.3 ± 1.0 lifetime partners (range: 1 – 7) and reported lifetime condom use of 11.8% (N = 328). One-fifth (23.1%) reported engaging the services of a female sex worker (FSW) and encounters occurred predominantly in Afghanistan (40.0%, 297/697; multiple answers allowed), Iran (30.7%, 214/697), and Pakistan (25.4%, 177/697). Of those reporting sex with FSWs, only 17.9% used a condom at the last encounter and 9.3% reported consistent (100%) condom use.

Sexual relations with adult men or boys were reported by 4.6% (N = 127) and 18.3% (N = 511), of whom 41.7% (N = 53) and 59.2% (N = 302) reported paying men or boys for sexual services, respectively. Few reported ever using condoms with men (7.9%) or boys (2.6%). Among those reporting sex with adult males, relations were largely described as insertive intercourse (79.5%), while 11.0% reported receptive intercourse only and 10.2% reported both. A regional distribution was noted among participants reporting MSM activity, with prevalence of 30% or more among sexually-active participants born in three northern provinces and among those born outside Afghanistan. While 1.6% (N = 75/4750) reported prior STI diagnosis, prior STI symptoms, comprising abnormal penile discharge, genital wart-like growths, and penile ulceration, were reported by 21.7% (N = 1029).

A sub-analysis was performed to assess correlates of sexual risk behaviors (e.g. MSM or sex with a FSW) among participants reporting prior sexual activity. In multivariable logistic regression controlled for province of origin, sex with FSWs was independently associated with reported use of condoms and alcohol, moving back to Afghanistan within the last year, television ownership, and greater number of prior sexual partners and age (Table 2). MSM activity was independently associated with prior use of hashish, penile discharge, and greater number of sex partners, while having lived outside Afghanistan and greater age remained negatively associated in multivariable analysis. Being married remained negatively associated with sex with FSWs and with MSM activity in multivariable analysis (Table 2).

**Table 2:** Correlates independently associated with sexual risk behaviors among sexually active male Afghan National Army recruits (N = 2786)

	**Risk group**	**Comparison group**		
**Variable**	**N, %**	**N, %**	**OR, 95% CI**	Adjusted OR, 95% CI*
*Relations with Female Sex Worker*	*(N = 593):*	*(N = 2193):*		
Age (years; mean, SD)	22.6, 3.8	23.2, 4.1	0.96, 0.94 - 0.99	
Lived outside Afghanistan	503, 84.8%	1546, 70.5%	2.34, 1.84 – 2.98	1.71, 1.26 – 2.31
Returned to Afghanistan within last year	261, 44.0%	526, 24.%	2.49, 2.06 – 3.01	1.60, 1.26 – 2.03
Years formal education	4.0, 4.1	3.5, 4.2	1.02, 1.01 – 1.05	
Currently married	204, 34.4%	1599, 72.9%	0.19, 0.16 – 0.24	0.21, 0.17 – 0.26
Television in home	256, 43.2%	627, 28.6%	1.90, 1.57 – 2.29	1.25, 1.00 – 1.56
Radio in home	445, 75.0%	1506, 68.7%	1.37, 1.11 – 1.68	
Eat meat ≥2 times weekly	113, 19.1%	304, 13.%	1.42, 1.15 – 1.86	
Prior incarceration	167, 28.2%	405, 18.5%	1.73, 1.40 – 2.13	1.32, 1.03 – 1.70
Prior depression/anxiety	215, 36.3%	712, 32.5%	1.18, 0.98 – 1.43	
Currently taking medication for depression/anxiety	16, 2.7%	68, 3.1%	0.87, 0.50 – 1.51	
Ever used alcohol	111, 18.7%	98, 4.5%	4.92, 3.67 – 6.57	2.10, 1.47 – 3.01
Ever used hashish/marijuana	195, 32.9%	421, 19.2%	2.06, 1.69 – 2.52	
Ever used opiates	55, 9.3%	88, 4.0%	2.45, 1.72 – 3.47	
Total lifetime partners (mean,SD)	1.9, 1.6	1.1, 0.60	2.04, 1.81 – 2.29	1.65, 1.47 – 1.85
Ever used condom	163, 27.5%	165, 7.5%	4.66, 3.66 – 5.92	3.15, 2.36 – 4.20
Ever have urethral discharge	159, 26.8%	495, 22.6%	1.26, 1.02 - 1.55	
Ever have genital warts/penile ulcers	5, 0.84%	28, 1.3%	0.66, 0.25 – 1.71	
*Sexual Relations with Male Partner*	*(N = 592):*	*(N = 2194):*		
Age (years; mean, SD)	21.3, 3.5	23.5, 4.1	0.84, 0.82 – 0.86	0.92, 0.89 - 0.96
Ever lived outside Afghanistan	413, 69.8%	1636, 74.6%	0.79, 0.64 – 0.96	0.56, 0.43 - 0.72
Lived outside Afghanistan until 1 year ago	161, 27.2%	626, 28.5%	0.94, 0.76 – 1.15	
Years formal education (mean, SD)	4.1, 4.2	3.5, 4.2	1.03, 1.01 – 1.05	
Currently married	161, 27.2%	1642, 74.8%	0.13, 0.10 – 0.15	0.17, 0.14 - 0.22
Television in home	202, 34.1%	681, 31.1%	1.15, 0.95 – 1.39	
Radio in home	428, 72.3%	1523, 69.4%	1.15, 0.94 – 1.40	
Eat meat ≥2 times weekly	111, 18.8%	306, 14.0%	1.42, 1.12 – 1.81	
Prior incarceration	127, 21.5%	445, 20.3%	1.07, 0.86 – 1.34	
Prior depression/anxiety	241, 40.7%	686, 31.3%	1.51, 1.25 – 1.82	
Currently taking medication for depression/anxiety	15, 2.5%	69, 3.1%	0.80, 0.45 – 1.41	
Ever used alcohol	63, 10.6%	146, 6.7%	1.67, 1.22 – 2.28	
Ever used hashish/marijuana	203, 34.3%	413, 18.8%	2.25, 1.84 – 2.75	1.86, 1.46 - 2.38
Ever used opiates	33, 5.6%	110, 5.0%	1.12, 0.75 – 1.67	
Total lifetime partners (mean, SD)	1.7, 1.4	1.2, 0.8	1.56, 1.43 – 1.70	1.45, 1.32 - 1.60
Ever used condom	68, 11.5%	260, 11.9%	0.97, 0.73 – 1.28	
Ever have urethral discharge	207, 35.0%	447, 20.4%	2.10, 1.72 – 2.56	1.86, 1.47 - 2.34
Ever have genital warts/penile ulcers	4, 0.68%	29, 1.3%	0.51, 0.18 – 1.45	

*Analysis adjusted for province of origin; only variables remaining in final model shown.

### Prevalence and correlates of infection

Low prevalence of HIV (0.063%, 95% CI: 0.013 – 0.185), syphilis (0.65%, 95% CI: 0.44 – 0.93), and hepatitis C (0.82%, 95% CI: 0.58 – 1.12) were detected and precluded further analysis due to power considerations. For HCV, of 43 reactive rapid test samples, 25 had HCV virus detected at PCR testing and thus were not confirmed for antibody per testing algorithm. Of those samples without detectable viremia, 14 had RIBA-detected HCV antibody, and four were negative for both virus and antibody (91% positive predictive value). Prevalence of HSV-2 was 3.03% (95% CI: 2.56 – 3.57); HSV-2 was positively associated with greater age, having a television in the home, prior alcohol use, and marginally with using a condom at last FSW encounter (Table 3).

**Table 3:** Correlates of herpes simplex-2 infection among a cross-sectional sample of male Afghan National Army recruits in univariable logistic regression, 2010–2011 (N = 4750)

**Variable**	**Infected (N,%)**	**Non-infected (N,%)**	**OR, 95% CI**	**AOR, 95% CI**
Age (years; mean ± SD)	22.5 ± 4.2	21.8 ± 3.8	1.04, 1.00 – 1.09	1.04, 1.00 – 1.09
Province of origin:			1.00, 1.00 – 1.01	
Nangahar	17, 11.8%	752, 16.3%		
Non-Nangahar	127, 88.2%	3854, 83.7%		
Education (years; mean ± SD)	4.46 ± 4.38	4.14 ± 4.33	1.02, 0.98 – 1.06	
Radio in the home	107, 74.3%	3181, 69.1%	1.29, 0.89 – 1.89	
Television in the home	61, 42.4%	1491, 32.4%	1.53, 1.10 – 2.15	1.46, 1.02 – 2.05
Lifetime depression/anxiety	39, 27.1%	1341, 29.1%	0.90, 0.62 – 1.31	
Prior alcohol use	14, 9.7%	235, 5.1%	2.00, 1.14 – 3.53	1.69, 0.95 – 3.01*
Prior hashish use	30, 20.8%	738, 16.0%	1.38, 0.92 – 2.08	
Prior opiate use	8, 5.6%	157, 3.4%	1.67, 0.80 – 3.46	
Prior incarceration	30, 20.8%	750, 16.3%	1.35, 0.90 – 2.04	
Number lifetime sexual partners (mean ± SD):				
Female	1.37 ± 1.20	1.28 ± 0.96	1.09, 0.92 – 1.30	
Male	0.19 + 0.72	0.18 + 0.59	1.03, 0.79 – 1.34	
Ever sex with FSW	21, 14.7%	572, 12.5%	1.21, 0.75 – 1.94	
Condom at last FSW encounter	7, 33.3%	99, 17.3%	2.33, 1.06 – 5.10	
Ever sex with another male	17, 11.9%	576, 12.6%	0.94, 0.56 – 1.57	
Lifetime condom use	17, 11.9%	311, 6.8%	1.86, 1.10 – 3.12	

OR = Odds ratio.

CI = Confidence Interval.

SD = standard deviation.

FSW = female sex worker.

*p = 0.07.

In multivariable models adjusted by province of origin, HSV-2 was independently associated with having a television, greater age, and marginally with prior alcohol use (Table 3).

## Discussion

To our knowledge, this study is the first to assess HIV or STI prevalence among a military population in Afghanistan, potentially a reflection of the general population, and one of few to describe STI prevalence among a nationally-derived sample [14-16]. Findings are notable for a very low prevalence of HIV and other STIs and for a small but sizeable proportion of incoming ANA recruits having engaged in sexual and drug use risk behaviors. Lifetime condom use is quite low and, coupled with reported sexual risk behaviors, may be one focal point within force health protection efforts.

Prevalence of HIV, syphilis, and HCV are quite low, similar to those measured among other populations of varying sexual risk (with the exception of injecting drug users) in Afghanistan [16,17]. Military recruits have been found to be an accurate general population proxy for prevalence of HIV and hepatitis C in some settings, but not others [1,18,19]. Lower rates of HIV have been detected among active duty troops as compared to the general population or military applicants in countries with compulsory HIV screening and regulations barring conscription of HIV-infected individuals [1,20]. Conversely, higher HIV prevalence has been detected among active duty troops than in general population groups, leading to the characterization of military populations as a high-risk group in some contexts [3,7,21]. With regard to risk behavior, some studies speculate that military populations have higher rates of risk behaviors, potentially decreasing ability to generalize prevalence of risk behaviors to a general population of young adults or have military populations serve as a sentinel group for risk behaviors [21]. The regular movement associated with postings and stress related to combat have been posited as reasons for military populations to engage in behaviors placing them at greater risk for STIs, including HIV [3,7,8,21]. Further research is needed to determine relative risk behaviors between military and non-military populations of young adults specific to each context.

HSV-2 prevalence was also relatively low and was not directly associated with risky sexual practices, but with age, having a television, and, marginally, prior alcohol use. The association between HSV-2 and increasing age likely reflects exposure time and has been noted in other military populations [5,22]. Television ownership was associated with both HSV-2 and sex with FSWs in this population and may represent peri-urban location and financial resources enabling purchase of sex. Higher socioeconomic status was a marker for HSV-2 infection among Chinese male migrant workers in the presence of no associated sexual behaviors [23]. HSV-2 was marginally independently associated with prior alcohol use, potentially representing unsafe sexual practices while intoxicated. Prior alcohol use was also independently associated with engaging FSW services; alcohol consumption, an illicit activity in Afghanistan, may have occurred with FSWs. Data from Afghan FSWs support this possibility as, of those who used alcohol or other intoxicants (9.8%), 53.9% used these substances with their clients [16]. Though HSV-2 was not associated with engaging FSW services, this activity may have been under-reported.

Reported relations with FSWs were also independently associated with prior condom use, previously living outside Afghanistan and having returned within the last year, prior incarceration, and higher number of lifetime sexual partners. Lifetime condom use was low among recruits; it is unclear whether condom use is driven by need for contraception or STI prevention. However, reported consistent condom use with FSWs and condom use at the last FSW encounter were relatively uncommon. Further, it is also unclear whether condoms used in the context of paid sex are at the behest of the client or FSW, as prior studies indicate that a majority of Afghan FSWs state they determine condom use with clients [24]. Motivations surrounding condom use require greater information in this population to inform prevention efforts. Having lived outside Afghanistan and recent repatriation were both associated with engaging FSW services, as noted among other male Afghan expatriates [25]. The association between incarceration and sex with FSWs is unclear but may be reflected by 30% of previous incarcerations occurring outside Afghanistan, despite being independent of living outside the country in analysis. Greater number of lifetime partners has not been directly associated with FSW patronage among military populations, but higher numbers of partners increased HIV acquisition risk in a context where HIV was largely attributed to unprotected sex with FSWs among male Thai military recruits [26].

MSM activity was common among sexually-active recruits and was associated with hashish use, history of urethral discharge, greater number of sexual partners, younger age, and never having lived outside Afghanistan. In Afghanistan, hashish is the most commonly used intoxicant and use among males begins at a young age (18–19 years) [27]. The association of MSM activity with young age, hashish use, and not having lived outside Afghanistan potentially reflects normative behaviors in certain areas of the country. Of note, though the analysis was controlled by province, province of origin was significantly associated with MSM activity. The association between MSM activity and number of sexual partners may reflect the paid nature of half of these relations and the association with prior cases of urethral discharge may reflect low condom use. Among Thai military conscripts, MSM activity was associated with hashish use, a greater number of sexual partners, and prior urethritis as compared to those reporting exclusively heterosexual contacts in the mid-1990s [28]. In our study, having a current marital partner was negatively associated with both sexual risk behaviors and may reflect both limited options for sexual activity for unmarried men and potential social stigma associated with adultery in this conservative setting. By contrast, engaging in MSM activities were more likely for married male military recruits in Thailand [28].

There are limitations to this study that must be considered. First, though participants were randomly selected during a 12-month period, approximately one-fifth originated from one province. Analyses were adjusted by province of origin to reduce this effect, but this disproportion should be considered during interpretation. The high participant number from Nangahar may be associated with two different factors: proximity to Kabul, enabling potential recruits to come to KMTC rather than recruitment through local garrisons, and instability in western Pakistan may have displaced Afghan families whose sons were employed or students in Pakistan, resulting in need for stable employment. We only obtained serum samples and cannot comment on the prevalence of STIs that typically cause genitourinary symptoms, such as *N. gonorrhea* or *C. trachomatis*. Correlations of reported genitourinary symptoms and likely presence of STIs should thus be interpreted with caution. Due to low literacy, questionnaires had to be administered by study staff, potentially reducing disclosure of sexual or drug use activities. Sex-matched study staff were trained extensively in counseling and ability to establish rapport to counter this effect. Last, certain provinces may have been under-represented, particularly provinces (Nuristan and Laghman) from which Pashayee speakers originate, as they comprised the bulk of those ineligible due to language differences.

## Conclusions

In conclusion, though prevalence of HSV-2, hepatitis C, and HIV are currently low among ANA recruits, the history of prior and potentially ongoing risk behavior is cause for concern. The ANA, like many other militaries, has a separate health care system. Preventive medicine programming is minimal and largely focuses on personal hygiene and vaccination at enlistment (personal communication, GEN J.M. Khalazai). Staff from the Religious Affairs department provide counseling and ensure processing of benefits to families of deceased or disabled soldiers. These staff also perform periodic visits and deliver message-based speeches based on religious text to promote nationalism and hygiene practices and do not include any messages regarding drug use or sexual behaviors currently. The recruit training period provides an important opportunity for a risk reduction intervention, noted to be effective in Nigeria and Angola [9,21]. The recruitment period afforded time for performance of this study that might be similarly utilized for a behavioral intervention. However, due to language and ethnic diversity within the ANA, further formative work is needed to ensure messaging is accurate and culturally-appropriate.

## Abbreviations

ANA: Afghan National Army; ANP: Afghan National Police; ANSF: Afghan National Security Forces; AOR: Adjusted Odds Ratio; CI: Confidence Interval; FSW: Female Sex Worker; HCV: Hepatitis C Virus; HCV Ab: Hepatitis C Virus Antibody; HIV: Human Immundeficiency Virus; HSV-2: Herpes Simplex 2 Virus; KMTC: Kabul Military Training Center; MoPH: Ministry of Public Health; MSM: Men having Sex with Men; OR: Odds Ratio; PCR: Polymerase Chain Reaction; RIBA: Recombinant Immunoblot Assay; RPR: Rapid Plasma Reagin; STI: Sexually-Transmitted Infection; TPPA: *Treponema Pallidum* Plasma Agglutination.

## Competing interests

The authors declare they have no conflicts of interest.

## Authors’ contributions

CST, RVB, and PTS developed the protocol, AN managed field activities and laboratory analyses, GFM and SMS supervised field data collection and GFM additionally supervised data entry and cleaning, LLJ provided laboratory support and assisted with data interpretation, BRH, FS and MNK provided input on protocol development with regard to pre-existing ANA policies and advised field implementation strategies, and CST performed data analysis and lead manuscript preparation. All authors reviewed and approved the final submission. CST confirms she has had full access to the data and the final responsibility for the decision to submit for publication.

## Funding

This study was funded by the Walter Reed Army Institute of Research. Material has been reviewed by the Walter Reed Army Institute of Research. There is no objection to its presentation and/or publication. The opinions or assertions contained herein are the private views of the authors, and are not to be construed as official, or as reflecting true views of the United States Department of the Army or the Department of Defense.

## Pre-publication history

The pre-publication history for this paper can be accessed here:

http://www.biomedcentral.com/1471-2334/12/196/prepub
